# Supplementary figures and images for: Structures of topoisomerase V in complex with DNA reveal unusual DNA-binding mode and novel relaxation mechanism
Source: eLife. 2022 Aug 15;11:e72702. doi: 10.7554/eLife.72702 (PMC9489208; doi:10.7554/eLife.72702)

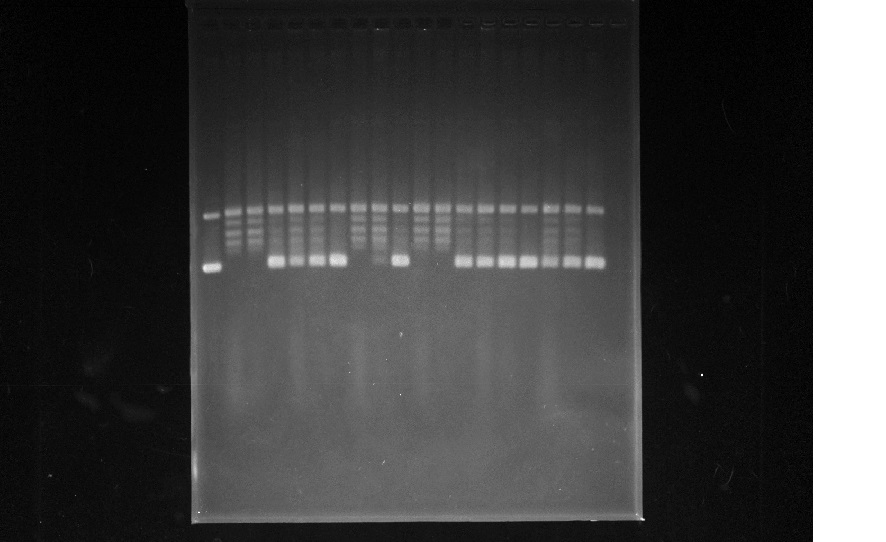

Supplement: Figure 4—figure supplement 1—source data 1. — The figure shows typical DNA relaxation assays for the wild-type protein and the Arg37Ala, Lys47Ala, His56Ala, Arg83Ala, and Ala132Ile mutants. For each relaxation assay, 0.15, 1.5, and 3.5 µg of the enzyme were used together with 306 ng of negatively supercoiled pUC19 plasmid (Materials and methods). Mutants are labeled and colored to reflect the assessed level of activity (green: wild-type level of activity, blue: reduced activity, orange: minimal activity, and pink: no activity). The location of supercoiled, relaxed, and nicked plasmid DNA is marked. [file elife-72702-fig4-figsupp1-data1.zip › Osterman-Mondragon-Figure4Supp1Column1-SourceData.jpg]

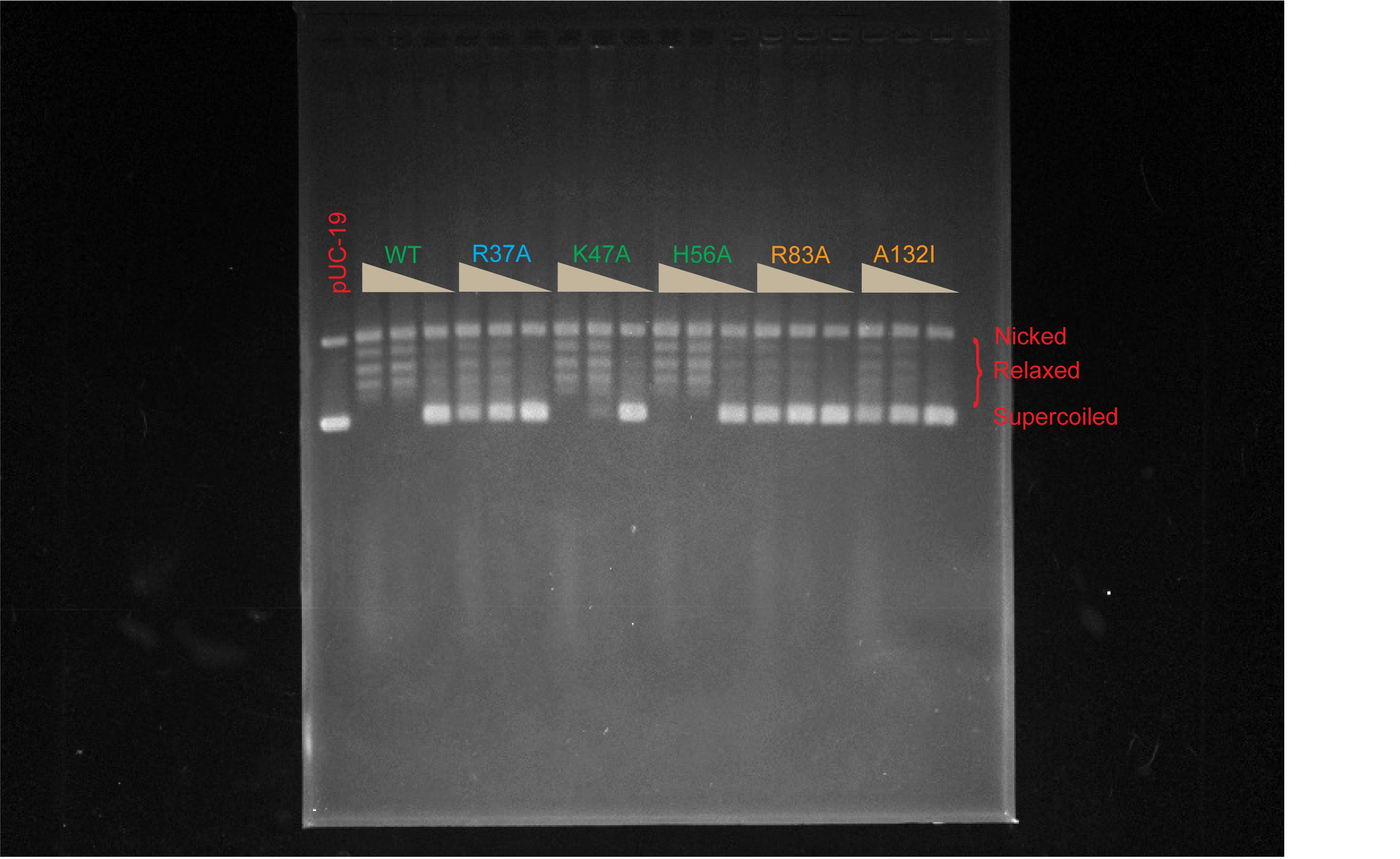

Supplement: Figure 4—figure supplement 1—source data 1. — The figure shows typical DNA relaxation assays for the wild-type protein and the Arg37Ala, Lys47Ala, His56Ala, Arg83Ala, and Ala132Ile mutants. For each relaxation assay, 0.15, 1.5, and 3.5 µg of the enzyme were used together with 306 ng of negatively supercoiled pUC19 plasmid (Materials and methods). Mutants are labeled and colored to reflect the assessed level of activity (green: wild-type level of activity, blue: reduced activity, orange: minimal activity, and pink: no activity). The location of supercoiled, relaxed, and nicked plasmid DNA is marked. [file elife-72702-fig4-figsupp1-data1.zip › Osterman-Mondragon-Figure4Supp1Column1-SourceDataLabeled.tif]

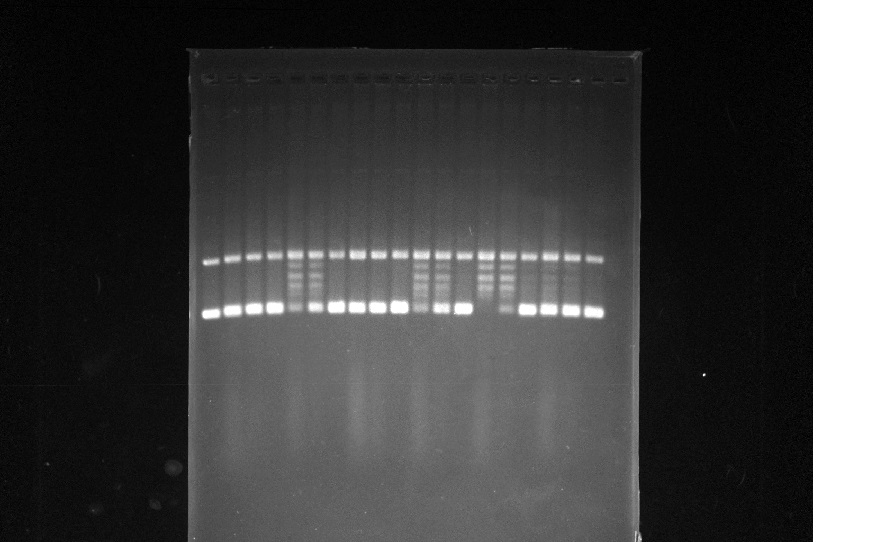

Supplement: Figure 4—figure supplement 1—source data 2. — The figure shows typical DNA relaxation assays for the Arg108Ala, Arg109Ala, Arg108Ala/Arg109Ala, Lys134Ala, Arg135Ala, and Lys134Ala/Arg135Ala mutants. For each relaxation assay, 0.15, 1.5, and 3.5 µg of the enzyme were used together with 306 ng of negatively supercoiled pUC19 plasmid (Materials and methods). Mutants are labeled and colored to reflect the assessed level of activity (green: wild-type level of activity, blue: reduced activity, orange: minimal activity, and pink: no activity). The location of supercoiled, relaxed, and nicked plasmid DNA is marked. [file elife-72702-fig4-figsupp1-data2.zip › Osterman-Mondragon-Figure4Supp1Column2-SourceData.jpg]

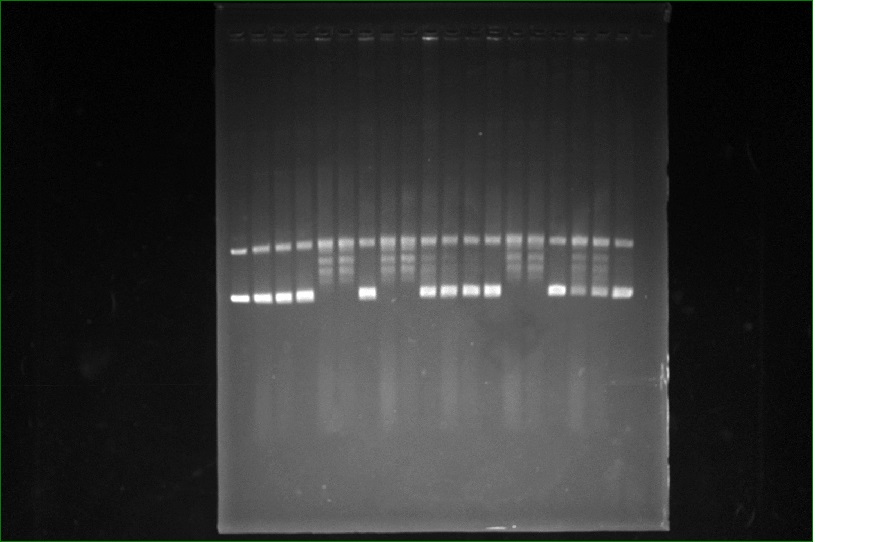

Supplement: Figure 4—figure supplement 1—source data 3. — The figure shows typical DNA relaxation assays for the Lys134Glu/Arg135Glu, Arg228Ala, Tyr289Ala, Leu290Pro, Arg293Ala, and Arg288Ala/Arg293Ala mutants. For each relaxation assay, 0.15, 1.5, and 3.5 µg of the enzyme were used together with 306 ng of negatively supercoiled pUC19 plasmid (Materials and methods). Mutants are labeled and colored to reflect the assessed level of activity (green: wild-type level of activity, blue: reduced activity, orange: minimal activity, and pink: no activity). The location of supercoiled, relaxed, and nicked plasmid DNA is marked. [file elife-72702-fig4-figsupp1-data3.zip › Osterman-Mondragon-Figure4Supp1Column3-SourceData.jpg]

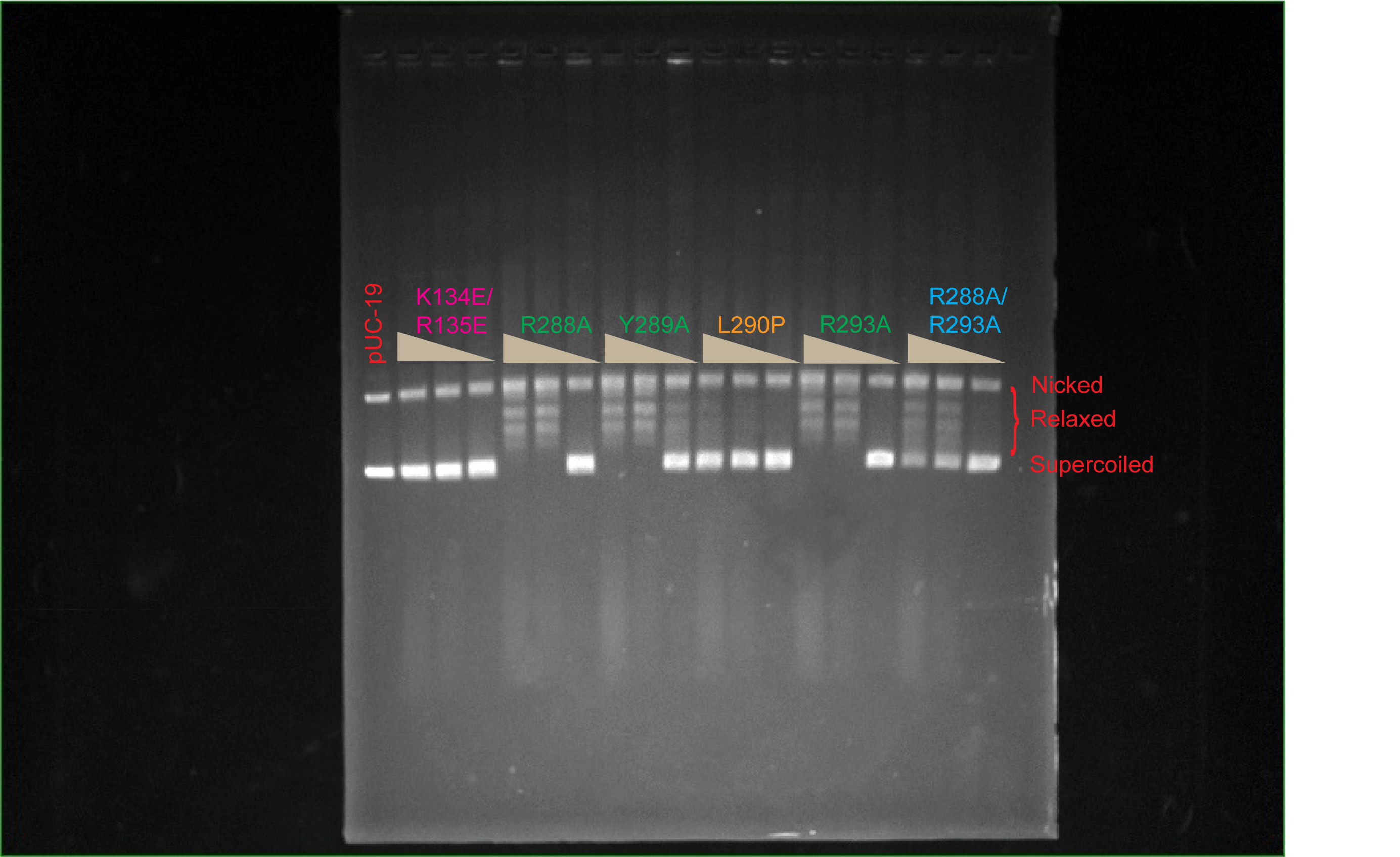

Supplement: Figure 4—figure supplement 1—source data 3. — The figure shows typical DNA relaxation assays for the Lys134Glu/Arg135Glu, Arg228Ala, Tyr289Ala, Leu290Pro, Arg293Ala, and Arg288Ala/Arg293Ala mutants. For each relaxation assay, 0.15, 1.5, and 3.5 µg of the enzyme were used together with 306 ng of negatively supercoiled pUC19 plasmid (Materials and methods). Mutants are labeled and colored to reflect the assessed level of activity (green: wild-type level of activity, blue: reduced activity, orange: minimal activity, and pink: no activity). The location of supercoiled, relaxed, and nicked plasmid DNA is marked. [file elife-72702-fig4-figsupp1-data3.zip › Osterman-Mondragon-Figure4Supp1Column3-SourceDataLabeled.tif]

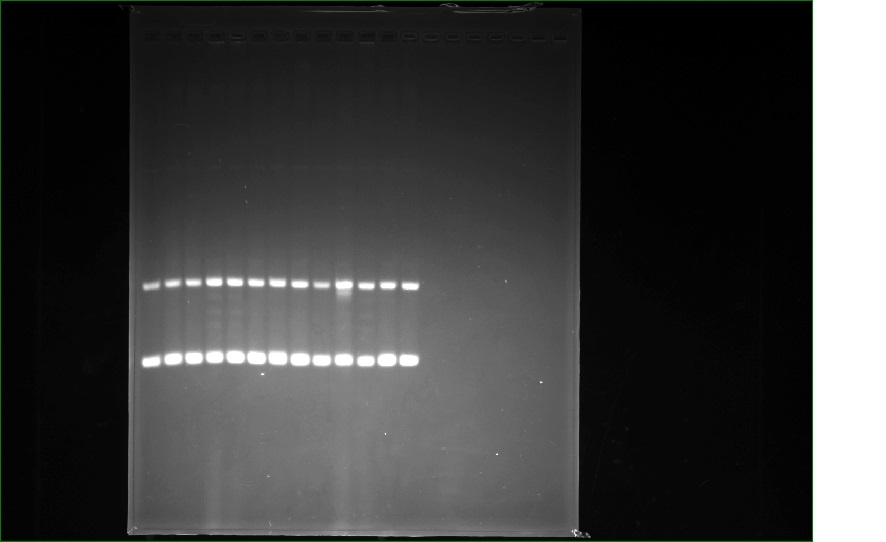

Supplement: Figure 4—figure supplement 1—source data 4. — The figure shows typical DNA relaxation assays for Arg288Glu/Arg293Glu and Arg288Glu/Leu290Pro/Arg293Glu mutants. For each relaxation assay, 0.15, 1.5, and 3.5 µg of the enzyme were used together with 306 ng of negatively supercoiled pUC19 plasmid (Materials and methods). Mutants are labeled and colored to reflect the assessed level of activity (green: wild-type level of activity, blue: reduced activity, orange: minimal activity, and pink: no activity). The location of supercoiled, relaxed, and nicked plasmid DNA is marked. [file elife-72702-fig4-figsupp1-data4.zip › Osterman-Mondragon-Figure4Supp1Column4-SourceData.jpg]

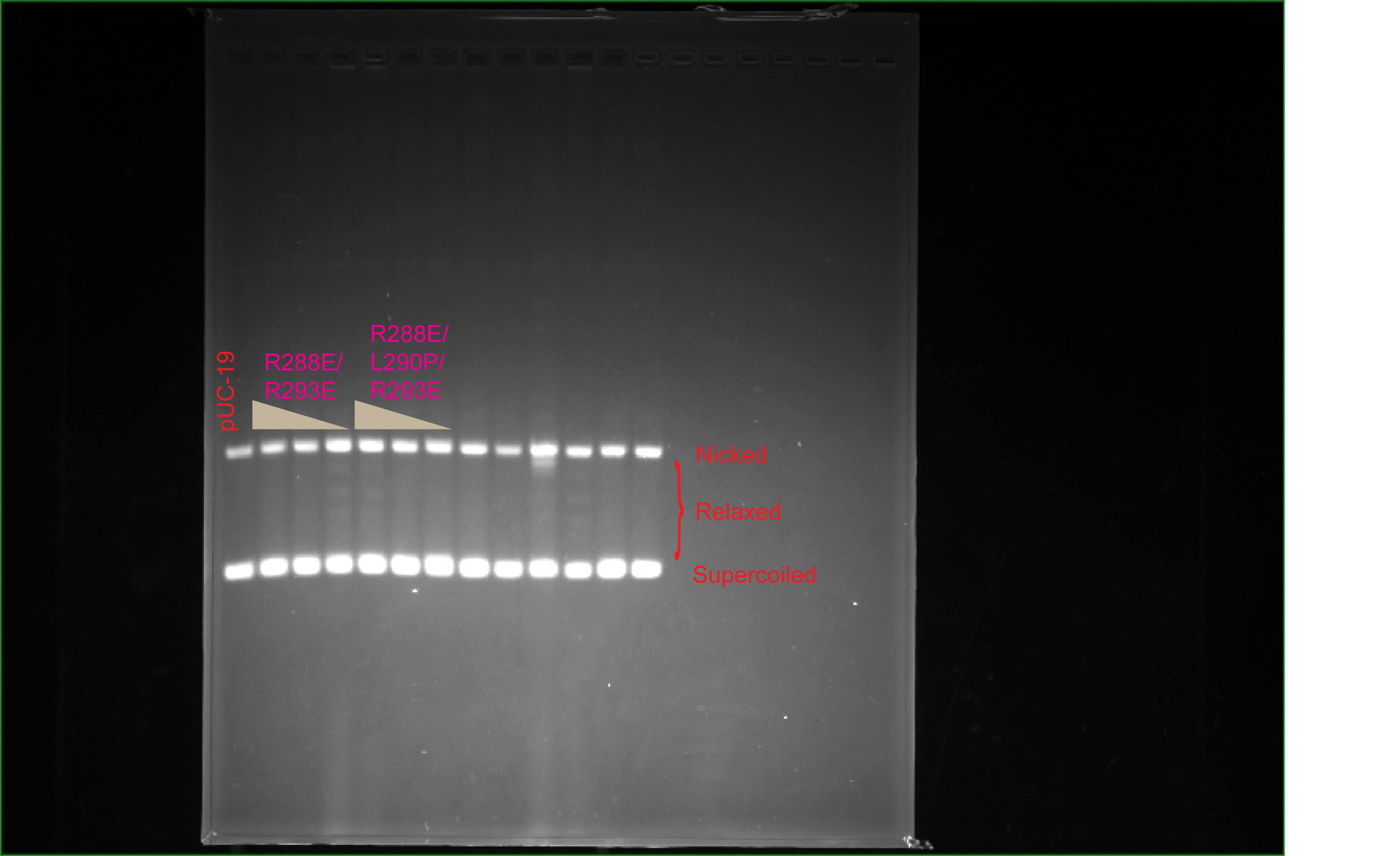

Supplement: Figure 4—figure supplement 1—source data 4. — The figure shows typical DNA relaxation assays for Arg288Glu/Arg293Glu and Arg288Glu/Leu290Pro/Arg293Glu mutants. For each relaxation assay, 0.15, 1.5, and 3.5 µg of the enzyme were used together with 306 ng of negatively supercoiled pUC19 plasmid (Materials and methods). Mutants are labeled and colored to reflect the assessed level of activity (green: wild-type level of activity, blue: reduced activity, orange: minimal activity, and pink: no activity). The location of supercoiled, relaxed, and nicked plasmid DNA is marked. [file elife-72702-fig4-figsupp1-data4.zip › Osterman-Mondragon-Figure4Supp1Column4-SourceDataLabeled.tif]
